# Leukocyte and platelet rich fibrin in the management of medication-related osteonecrosis of the jaw: A systematic review and meta-analysis

**DOI:** 10.4317/medoral.25733

**Published:** 2023-01-15

**Authors:** Ana Muñoz-Salgado, Fábio França Vieira e Silva, María Elena Padín-Iruegas, Gisela Cristina Vianna Camolesi, Wilber Edison Bernaola-Paredes, Henrique Rocha Mazorchi Veronese, Miriã de Andrade Celestino, William Jose Silva Filho, Alejandro I Lorenzo-Pouso, Mario Pérez-Sayáns

**Affiliations:** 1 Oral Medicine, Oral Surgery and Implantology Unit (MedOralRes), Faculty of Medicine and Dentistry, University of Santiago de Compostela, Santiago de Compostela, Spain; 2 ORALRES Group, Health Research Institute of Santiago de Compostela (FIDIS), Santiago de Compostela, Spain; 3 Human Anatomy and Embriology Area, Department of Functional Biology and Health Sciences University of Vigo, Lagoas-Marcosende, Vigo, Spain; 4 Department of Radiation Oncology, A.C. Camargo Cancer Center, Sao Paulo, Brazil; 5 Department of Stomatology, School of Dentistry, UNIFAMINAS, Minas Gerais, Brazil; 6 Dentistry Department, Federal University of Sergipe, Aracaju, Brazil

## Abstract

**Background:**

Osteonecrosis of the jaw (ONJ) has a frequent adverse effect after the administration of nitrogenous bisphosphonates, as non-nitrogenous bisphosphonates are metabolized more rapidly and would produce this effect to a lesser extent. The objective of this study is to analyze the results obtained in the literature with the use of L-PRF in the treatment of ONJ through a systematic review and meta-analysis.

Material and methods: Medline (via PubMed), Cochrane, Web of Science and Grey Literature Database was screened from which 10 were selected.

**Results:**

In the meta-analysis with full resolution, combining the use of L-PRF in the treatment of ONJ, a weighted proportion (PP) of 94.3% of complete resolution is obtained (95% CI: 91.2-97.4, *p*<0.001), with a low degree of heterogeneity, statistically significant (I2 = 29.02%; *p*<0.001). When analyzing the non-resolution data, a weighted proportion (PP) of 7.7% (95% CI: 3.6-11.9; *p*<0.001) was obtained with moderate heterogeneity (I2: 41.87%; *p*=0.112). In the meta-regression, no significant correlation was found between complete resolution and year of publication (intercept = 2.88, *p*=0.829). In consistency analysis no major changes in PP are identified when any of the studies are eliminated, demonstrating a high reliability in the combined results.

Conclusion: L-PRF alone or in combination with other therapies in treatment of ONJ achieved high percentages of complete lesion resolution (94.3%). In studies where L-PRF is combined with other therapies, and where the effectiveness of the other therapy alone is analyzed, L-PRF has been shown higher percentages of resolution.

** Key words:**L-PRF, osteonecrosis, treatment, bisphosphonates, monoclonal antibody.

## Introduction

Osteonecrosis of the Jaw (ONJ) has been recognized since the beginning of the century as a frequent adverse effect after the administration of certain drugs, although the term used to describe it has changed over the years (1). This term mainly refers to the use of nitrogenated bisphosphonates, because non-nitrogenated bisphosphonates have a faster metabolism and this effect would be produced in a lower way (2,3). The action mechanism is produced by acting on osteoclasts, their formation, differentiation, and function; these produce a lower bone remodeling, an increase in bone mineral density and a reduction in vertebral and long bone fractures (4). The presence of this disease only in the jaws has been associated with specific environmental factors in this area, for example, stress situations or cellular infection and subversion of the inflammatory response in the oral region, such as the presence of teeth with periodontal disease, infectious pathology and endodontic treatment. In addition, contrary to the rest of the bones, these have greater vascularization and a high rate of bone resorption (3,4).

Over the years, the definition of the disease has been changing, as it has been recognized that it also appears not only in association with BP but also with other antiresorptive therapies. Monoclonal antibodies treatment, some of them with an antiangiogenic effect (5). For this reason, the term Medication-Related Osteonecrosis of the Jaw (MRONJ) was introduced. The American Association of Oral and Maxillofacial Surgeons (AAOMS) defines that a case of MRONJ should include all the following elements: previous or current treatment with antiresorptive therapy alone or in combination with immune modulators or antiangiogenic medications, bone exposed or that can be probed through an intraoral or extraoral fistula(e) in the maxillofacial region that has persisted for more than eight weeks and without a history of radiation therapy or metastatic disease to the jaws (5).

Prevention has become an essential factor in the treatment of this disease. During the treatment with antiresorptive or antiangiogenic drugs the underlying systemic pathology should be sTable. Smoking cessation, regular visits to the dentist and a correct oral hygiene should be promoted (4). Regarding dental surgical procedures, high-risk procedures should be performed before starting therapy, using antibiotics and antiseptics, and performing primary wound closure. In relation to the cessation of antiresorptive and antiangiogenic drugs before extractions or other invasive dental procedures, it still produces controversy (5,6).

The incidence of MRONJ varies according to the type of study and the drug used. For example, in cases of non-nitrogenated BP the incidence is lower and progressively increases with the potency of the BP increment (7,8). In nitrogenated BP, the prevalence increases with the treatment time increment. In the cases of drugs used in cancer treatment, the incidence is significantly higher (9).

Different clinical disease levels have been established determined by extension, severity and symptoms (5,9). Moreover, there are many treatment possibilities, from a clinical approach with antibiotic and antiseptic methods, oral hygiene and conservative interventions, like bone spicule debridement in slight cases, to higher bone debridement alone or joined with other intervention modalities such as platelet concentrates, hormone therapy, ozone therapy, hyperbaric oxygen therapy or laser (4,5,10-12). Leukocyte and Platelet Rich Fibrin (L-PRF) is an autologous platelet concentrate obtained by blood extraction and without the addition of other substances. The basis of this compound is thrombocytes, leukocytes and stem cells, which produce a fibrin with a three-dimensional matrix capable of releasing growth factors, cytokines and proteins related to the recovering of the lesion, promoting cell proliferation and differentiation (13,14). The release of growth factors and membrane proteins happens for more than 7 days, producing the correct wound-healing of the lesion. The procedure consists of the extraction of consecutive blood tubes, depending on the patient's necessities and the size of the lesion, and their immediate centrifugation. Several studies have confirmed its efficacy, alone or in combination with other therapies, in the treatment of MRONJ (13).

Therefore, the aim of this study is to analyze the results obtained in the literature with the use of L-PRF in the treatment of ONJ through a systematic review and meta-analysis.

## Material and Methods

The protocol of this systematic review was previously designed by MPS and WEBP and agreed upon by all authors and registered in PROSPERO (CDR42021238864). The present systematic review and meta-analysis was performed according to Preferred Reporting Items for Systematic Reviews and Meta-Analyses (PRISMA), and the guidelines of the Meta-analysis of Observational Studies in Epidemiology (MOOSE) group.

The search question was formulated according to the PICO framework, and it read as follows: “What is the effectiveness of L-PRF application in the treatment of MRONJ?” According to the PICO method: population (patients with MRONJ), intervention (the L-PRF application in the treatment of MRONJ), comparison (those subjects that do not reach wound-healing with the application of L-PRF), outcome (effectiveness of the L-PRF treatment in terms of curation/improvement from baseline).

- Search strategy

Medline (via PubMed), Cochrane and Web of Science was screened. Grey Literature Database was screened at the New York Academy of Medicine Grey Literature. The last bibliographic search was carried out on April 4, 2022. Searches were conducted by combining thesaurus terms used by the databases (e.g., MeSH and EMTREE) and free text words. The search syntax was as follows: (("L-PRF" [All Fields] OR "leucocyte and platelet-rich plasma" [All Fields] OR "PRF" [All Fields]) AND ("osteonecrosis" [All Fields] OR "osteonecrosis of the jaw" [All Fields] OR "bone necrosis" [All Fields] OR "jaw necrosis" [All Fields] OR "ONJ" [All Fields] OR "BRONJ" [All Fields] OR "MRONJ" [All Fields] ) OR "ARONJ" [All Fields])).

The search strategy was combined with manual search in journals related to oral medicine, oral/maxillofacial surgery, oral pathology and oncology: Anticancer Research; Oral Biology Archives; Clinical Oral Investigations; European Archives of Otorhinolaryngology; European Journal of Oral Sciences; Neck; International Journal of Oral and Maxillofacial Surgery; International Journal of Oral Science; Journal of Craniomaxillofacial Surgery; Journal of Oral and Maxillofacial Surgery; Journal of Oral Pathology and Medicine; Oral Diseases; Oral Oncology; Oral Surgery, Oral Medicine, Oral Pathology and Oral Radiology. Potentially relevant articles with which any of the authors were familiar, as well as the reference lists of retrieved articles, were also comprehensively checked.

All references retrieved were managed using the software Mendeley (Elsevier, London, UK) and duplicated references were eliminated with this digital utility.

- Eligibility criteria

An ad hoc review team was composed to carry out this systematic review. This team was composed by two specialists in oral medicine/pathology (MPS and WEBP). The articles were selected in three phases by authors, first screening by titles, then reading the abstracts and, as a last phase, reading the entire text for inclusion. During the calibration exercise, the reviewers discussed the criterion and applied it to 50% of retrieved articles to determine inter-examiner agreement. After reaching an agreement, a kappa index (k) of 0.79 (95% CI 0.92-0.65) was established. Discrepancies between the two authors were resolved by a third author.

Inclusion criteria: i) patients with a clinical diagnosis of MRONJ; ii) use of L-PRF therapeutically, alone or in combination with other therapies; iii) surgical protocol correctly and exhaustively described; iv) publication in English.

Exclusion criteria: i) animal studies; ii) systematic reviews, case series of less than three cases, and letters to the editor; iii) unavailability of the full text.

- Data extraction

Data collected (Table 1, Table 2) were: first author, year of publication, country, study design, sample size, mean age, gender of the participants, previous pathology, local etiology, maxilla and mandible locations, type of medication administered, time of administration, medication discontinuation, MRONJ classification, MRONJ stage, surgical protocol, L-PRF preparation, follow-up of study participants, complete resolution, partial resolution, and no resolution.

- Quality assessment

JBI scale checklist: This method was used for prevalence studies. It is a qualitative analysis with 9 sections where it is evaluated the appropriating of the sample frame to address the population, the way to sampling the study participants, the adequation of sample size, the description in detail of the study subjects and the setting, the performing with sufficient coverage of the sample data analysis, the employing of valid methods for the identification the condition, the measuring of the variable in a standard and reliable way for all participants, the appropriability of statistical analysis and the adequacy of the response rate and the management of low response rates. Each domain is categorized as "Yes" (low risk of bias; +), "Not clear" (moderate risk of bias; ¿), "No" (high risk of bias; -), or not applicable. Studies were classified as high risk of bias if there was less than 49% "Yes", moderate risk of bias if the proportion "Yes" was 50 to 69%, and low risk if there was more than 70% "Yes".

The Newcastle-Ottawa Scale (NOS): This method is usually used for assessing the quality of non-randomized studies and has adapted for cohort and case-control studies. Three dimensions are measured on this scale: selection, comparability of cohorts and outcomes. In selection, four items are measured: representativeness of the cohort, selection of the unexposed cohort if any, exposure verification, and demonstration that the outcome of interest was not present at baseline. In comparability, the similarities between the cohorts are measured both in study design and analysis. In results, three items are measured; how the evaluation of the result has been done, the follow-up time and the adequacy of the follow-up of the cohorts. Each item can be rated with zero, one or two stars maximum (in the comparability section). In the analysis, studies rated 1-3 stars were defined as low quality, those rated 4-6 stars as medium quality, and 7-8 stars as high quality.

Cochrane Collaboration's tool: This method was used for randomized clinical trials and consisting of six domains: selection bias, performance bias, detection bias, attrition bias, reporting bias, and other biases (in this case, comparability of studies). Each of the domains was scored as ''low risk'' of bias (+), ''high risk'' of bias (-), or ''unclear'' risk of bias (?). Studies were classified as low risk if there was low risk of bias in all domains, as unclear risk of bias if there was low or unclear risk of bias in all domains; or high risk of bias if there are one or more domains with high risk of bias (15).

Risk of Bias in Non-Randomized Studies of Interventions (ROBINS-I) assessment toll: This method was used for non-randomized clinical trials. This scale is composed by seven domains: bias due to confounding, bias in the selection of study participants, bias in the classification of interventions, bias due to deviations from the desired intervention, bias due to missing data, measurement bias of the results, bias in the selection of the results reported. Each item is rated as low, moderate, serious or critical risk of bias or, finally, that there is no information to judge whether there is a risk of bias. The interpretation of each study is performed based on a Table, where the study is classified depending on the level of bias of each domain. Studies that have low risk in all domains are qualified with low risk of bias. Studies in which there is at least one domain with moderate risk of bias are rated as moderate risk of bias. Studies that have at least one domain with serious or critical risk of bias are scored, respectively, as having serious or critical risk of bias (16).

**Table 1:**
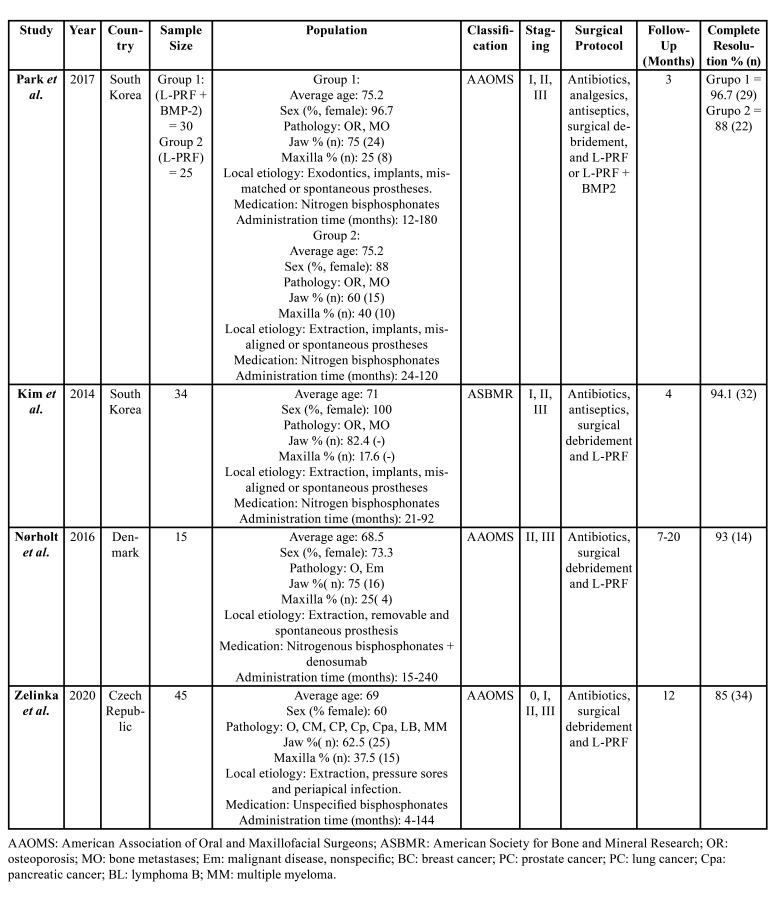
Data extraction randomized and non-randomized clinical trials.

**Table 2:**
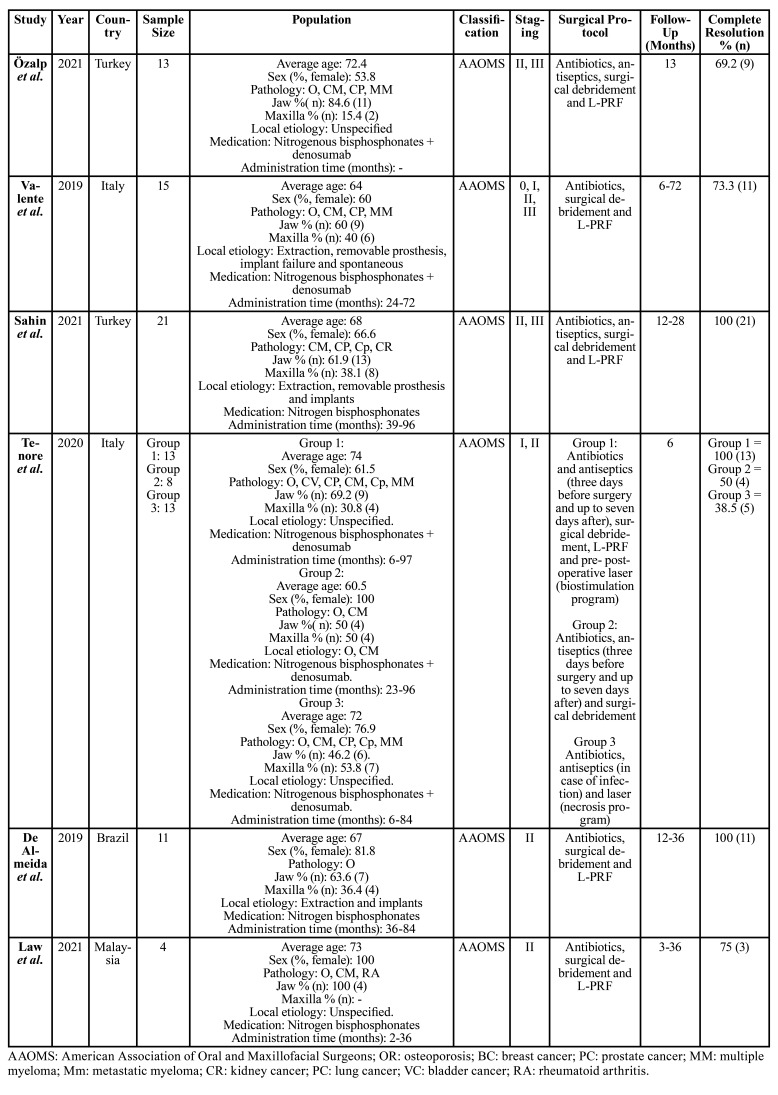
Data extraction case series and cohorts.

- Statistical analysis

In the main meta-analysis, we calculated the resolution rate of MRONJ cases treated with L-PRF by dividing the number of events by the study sample size. After that, we weighted the study-specific prevalence by the inverse of its variance to calculate a pooled prevalence. For the retrieved studies, the Clopper-Pearson interval was applied to estimate the 95% confidence intervals (CIs).

Due to the difficult comparison between the treatment arms due to the different surgical protocols in the cohort studies and clinical trials, the performance of meta-analyses with other effect measures to infer association, such as the odds ratio or the relative risk, was ruled out. At the same time, after a post hoc analysis, it was decided not to perform an analysis in subgroups due to the high consistency of the results.

Pooled analyzes were obtained using fixed effects models (Mantel-Haenszel method) and random effects models (DerSimonian and Laird method), but when considerable heterogeneity was detected, the assessment was based on random effects models only. An analysis via meta-regression was proposed to evaluate a covariate that applied to all the studies (year of publication), to know if a plausible improvement of the surgical protocols executed resulted in a positive intercept in the resolution rate.

For the analysis of statistical heterogeneity, the Cochran Q (χ2) and Higgins I2 (17) test parameters were calculated. Cochran's Q test *p* < 0.1 was considered significant to assume apparent heterogeneity. Higgins I2 statistic cutoff points of 25, 50, and 75% were considered to indicate low, moderate, and high heterogeneity, respectively (17).

We assessed publication bias, first visually, using funnel plots, and then, more formally, using the test proposed by Egger *et al*. (18) (performing a linear regression of the effect estimates on their standard errors, weighting by 1/[variance of the estimate of the effect], considering significant a pEgger < 0.1).

The R Metafor software package (v.3.6.2; https://www.r-project.org) was used for all statistical analyses, as well as for plotting Figures based on commands previously written by the user (19). The level of significance considered in all statistical analyzes was 5% (*p* < 0.05).

## Results

- Study selection process and study features

**Figure 1:**
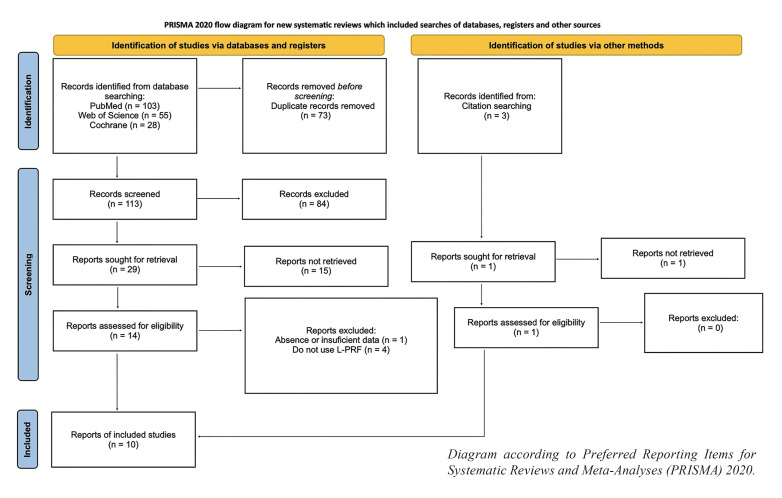
Flow diagram of literature search.

The selection of articles was reflected in the following image (Fig. 1). Using the search criteria described above, PubMed yielded 103 studies, Cochrane yielded 28, and Web of Science yielded 55.

After removing duplicates, 113 studies were retrieved. After filtering by titles, 29 were selected for abstracts complete review. Case reports of less than three cases, systematic reviews and those that did not use L-PRF as treatment were eliminated. After reviewing the abstracts, 14 were selected for full reading. Finally, of these 10 (0.08%) were selected. Of the 14, 3 were excluded because they used PRF or A-PRF and 1 was excluded because it was a series of cases in which only one case underwent treatment with L-PRF.

The main characteristics of the studies that met the eligibility criteria were collected (Table 1, Table 2). The geographic areas were: Korea (20,21), Denmark (22), Czech Republic (23), Turkey (24,25), Italy (26,27), Brazil (28) and Malaysia. Of the ten studies; four were cohort studies (24-27), two case series (28), one randomized clinical trial (20) and the rest non-randomized clinical trials (21-23). The publication dates ranged from 2014 to 2021.

All the studies except the one by Kim *et al*. (21) (which uses the classification of the American Society for Bone and Mineral Research, ASBMR), use the AAOMS classification to define the stage of MRONJ. The size of the samples varies from 4 to 40 patients.

In total, 196 patients diagnosed with MRONJ and treated with L-PRF were analyzed (20-28). The majority gender in all studies was female, finding a percentage of women between 53.8 and 100 (20-28). The mean age of the patients was between 60.5 and 75.2 years. The pathologies for which antiresorptive or antiangiogenic medication is prescribed are mainly: breast, gallbladder, prostate, lung, kidney, pancreas tumors, hematological diseases, rheumatoid arthritis and osteoporosis. The administration time of this medication ranged between 4 and 240 months (20-28). As a local etiology, the majority of studies include dental extractions, removable prostheses and dental implants (20-22,25,26,28). In all studies, the main location of MRONJ was the mandible, with percentages between 60 and 100% (20-28). In the study by Tenore *et al*. (27), the control groups (1 and 2) had a greater location of MRONJ in the maxilla than in the mandible, with Figures of 50 and 53.8%, respectively.

Only one study did not describe the type of BP administrated in patients. In the others, the predominant type of antiresorptive or antiangiogenic medication was nitrogenated bisphosphonates alone (20,21,25,28), followed by their combination with denosumab. The administration time of this type of medication varies between 2 and 240 months. Only three studies collect the drug holiday of medication before the treatment of MRONJ (22,25,28). Sahin *et al*. (25) ceased the medication between 2 and 10 months before, while de Almeida *et al*. (28), 3 months before in all participants. In the study by Nørholt *et al*. (22), it does not specify when, but it does specify that of the 15 participants, the medication was discontinued in 9.

The surgical protocol of most of the studies was first antibiotic therapy, with or without express indication of antiseptics use, debridement of necrotic area and application of L-PRF either in membranes or in plugs (20-28). One study (20) uses in addition BMP-2 while two others (25,27) perform laser therapy. Six of the studies use the Choukroun protocol by which 10 ml of venous blood is obtained from the patient and centrifuged at 3000 rpm for 10 minutes or at 2700 rpm for 12 minutes (20,21,24,25,27,28). The rest of the authors use other protocols: 1300 rpm for 10 minutes, 1300 rpm for 14 minutes (22) and 3200 rpm for 10 minutes (23). Only in one study the L-PRF preparation mode is not specified (26).

The follow-up was for at least 3 months, reaching even 72 months in some studies. In all studies, complete resolution was understood as healing or coverage of the entire lesion, without presenting clinical signs of MRONJ (20-28). Complete resolution percentages in patients who received L-PRF ranged from 69.2 to 100% in cohort studies and case series (24-28) and from 85 to 96.7% in clinical trials (20-23). The majority of authors conclude that L-PRF provides a great benefit in the treatment of MRONJ, at a low cost and with easy handling.

- Meta-analysis full resolution

**Figure 2:**
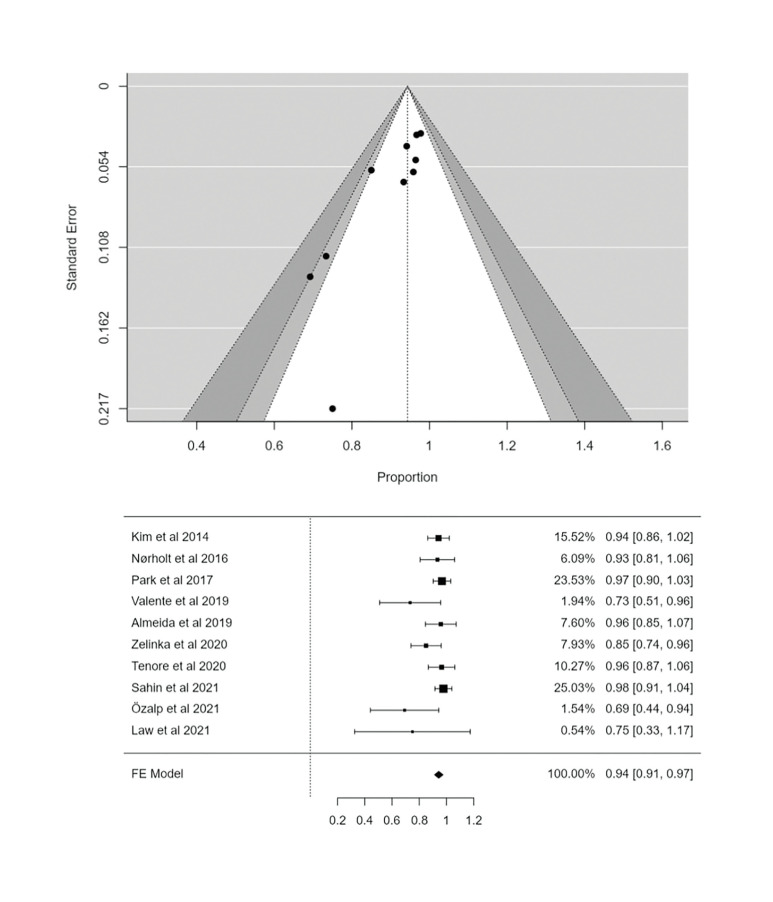
Forest plot and Funnel plot full resolution.

**Figure 3:**
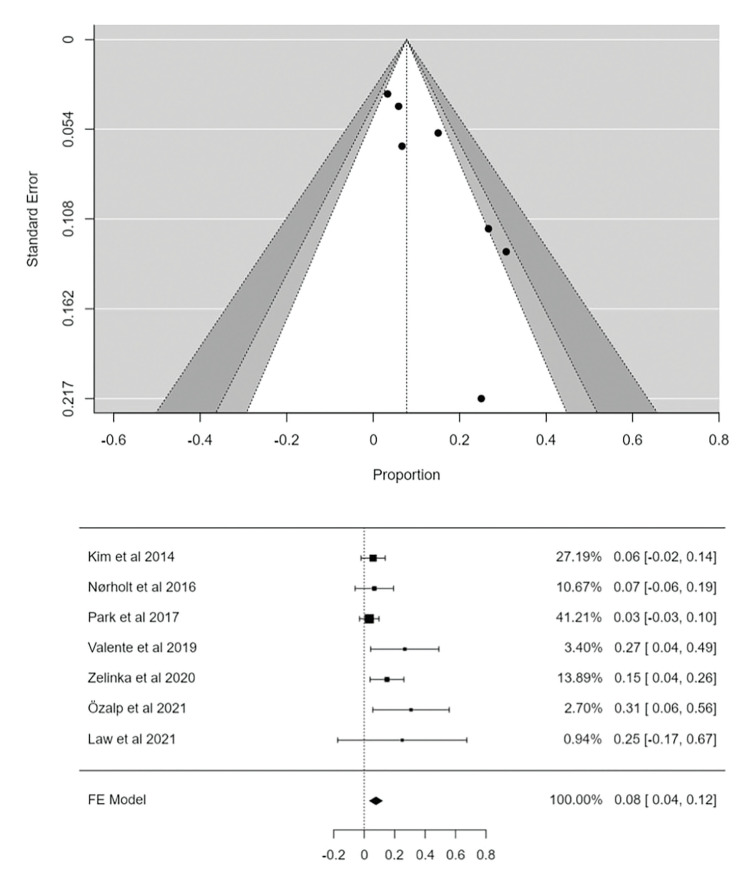
Forest plot and Funnel plot no resolution.

In the meta-analysis, combining the use of L-PRF in the treatment of MRONJ, a weighted proportion (PP) of 94.3% of complete resolution were obtained (95% CI: 91.2-97.4, *p*<0.001) (Fig. 2), with a low degree of heterogeneity, statistically significant (I2 = 29.02%; *p*<0.001) (Table 3). The funnel plot (Fig. 3) indicated quite a lot of symmetry with a slight deviation to the left, confirmed by Egger's test (PEgger = 0.178), which showed the existence of publication bias.

- Meta-analysis no resolution

In the analysis of non-resolution data, a weighted proportion (PP) of 7.7% (95% CI: 3.6-11.9; *p*<0.001) were obtained with moderate heterogeneity (I2: 41.87%; *p*=0.112) (Table 3). The funnel plot (Fig. 3) indicated asymmetry with a slight deviation to the right, also confirmed by Egger's test (PEgger = 0.112).

- Meta-regression

**Figure 4:**
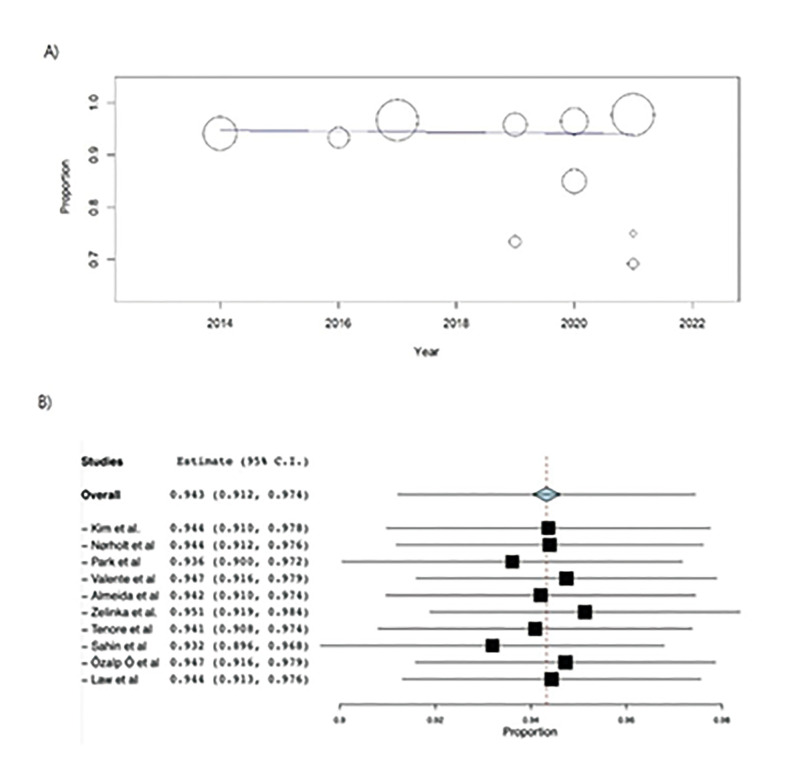
(A) Full-resolution meta-regression. (B) The Forest plot leave one out.

In the meta-regression, no significant correlation were found between the complete resolution and the publication year of the studies (intercept = 2.88, *p*=0.829) (Fig. 4).

- Consistency analysis

A consistency analysis has also been carried out, analyzing the influence of each study on the estimation of the general effect, checking the reliability of the combined results (29). Following the example of Viechtbauer (29), the meta-analysis has been repeated sequentially, omitting one study at a time and representing the results in the following graph (Fig. 4). No major changes in PP were identified when any of the studies were eliminated, so the high reliability in the combined results has been verified.

**Table 3:**
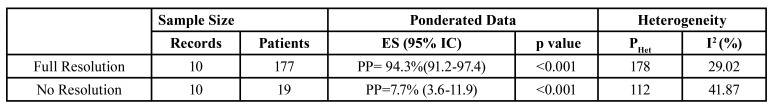
Weighted prevalence and heterogeneity analysis.

## Discussion

MRONJ is a serious, disabling disease, with a great impact on the life quality of patients. Its incidence among the population is low, with an estimated frequency in cancer patients of approximately 1% (range 0.2-6.7%), while in patients with osteoporosis receiving low doses of medication the incidence is 0.1% (range 0.004-0.2%) (30-33). There are numerous alternative therapies to L-PRF in the treatment of MRONJ, however, the great challenge of the current scientific society is to discover a standardized and safe treatment with high success rates. In this sense, we consider clinical success to achieve the complete remission of the disease, with wound closure of the lesion and, over the years, this has been achieved in different ways (3,5,11,33,34). The large heterogeneity of the disease and the different clinical stages have led to the appearance of numerous treatment therapies, from more conservative approaches in early stages to more radical treatments in advanced stages (3,5,11,33,34). According to the AAOMS, ASBMR and other scientific societies, the ideal treatment for MRONJ was a conservative approach, which included the use of antibiotics, antiseptics and debridement of the lesion only if it is considered necessary (5,34). However, the fact that on many occasions these injuries do not improve or even aggravate the injury has led to the search for alternative therapies (20,27,31). Among them, the one that currently seems to provide the most benefits is the use of L-PRF as an adjuvant to the protocol proposed by the AAOMS (11,13,28,20-27).

According to most authors, the low incidence and magnitude of the disease limits the existence of high-quality scientific publications such as randomized controlled clinical trials, with an adequate sample size, which would provide more scientific evidence (20-28).

Regarding the quality analysis of selected studies, according to the JBI scale, only one of the studies, Özalp *et al*. (24), presented a high risk of bias. The studies by Kim *et al*. (21), Nørholt *et al*. (22), Zelinka *et al*. (23), Valente *et al*. (26) and Almeida *et al*. (28) were classified as moderate risk and those by Tenore *et al*. (27), Park *et al*. (20), Sahin *et al*. (25) and Law *et al*. as low risk (Supplement 1; Supplement 2). Other scales were also used depending on the type of study. The NOS used in the four cohort studies and the case series, indicated that five of the studies had medium quality (24-28) and one high quality (27) (Supplement 3). The Cochrane collaboration's tool (15) was used in the randomized controlled study (20), indicating that it had an unclear risk of bias (since in half of the sections the risk of bias was unclear, and in the other half there was a low risk of bias) (Supplement 4). The ROBINS-I (16) was used in non-randomized trials, although only one intervention was performed on the study group, finding two studies with moderate risk (21,23) and one with serious risk of bias (22) (Supplement 5). After classifying the risk of the studies according to different scales, it is concluded that the most complete, adjusted to all the articles and most useful in this study was the JBI Checklist for prevalence studies, followed by the NOS scale.

Within the limitations of this study, it must be considered that it has only been possible to select a randomized clinical trial, but none controlled. The studies that have contributed the most to the weight of the meta-analysis according to the full resolution Forest Plot have been, in descending order, that of Sahin *et al*. (25), Park *et al*. (20), Kim *et al*. (20) and Tenore *et al*. (27). The meta-analysis has indicated, both in the case of resolution and non-resolution, a low degree of heterogeneity between the studies according to the pre-established cut-off points, both for the Higgins I2 test and for the *p-value* surrogate to the Cochran's Q test (Table 3).

The presence of the effect of publication biases in the proportion of the success rate analyzed should be considered. This method might lack of statistical power because the number of primary studies was limited to ten (35). In addition, previous simulation analyses point to a high rate of type 1 errors (i.e., false positives) when evaluating publication bias in meta-analyses of proportion studies with unusual events such as this one (15). Both the visual and statistical analyzes did not allow to rule out this publication bias (i.e., the tendency to publish only positive results) that is common in the health sciences literature and particularly in the field of surgery. Despite the above limitations, the robust nature of our meta-analyses is noTable in forest plot, showing powerful statistical associations, as well as by consistency analysis.

In this systematic review and meta-analysis in which various types of studies have been included (randomized and non-randomized clinical trials, cohorts and case series), the weighted prevalence of recovery after the use of L-PRF therapy was 94.3%. The results obtained are similar to those of the study by Di Fede *et al*. (34), where they report a success rate of 95% when combining surgery with the use of PRF, PRP or L-PRF. There are no more meta-analyses in the literature that analyze this association, so the comparability of the results obtained is limited. Performing only surgical debridement without other complementary techniques reaches a prevalence of total coverage, according to the data collected in the study by Di Fede *et al*. (34), of 81% at six months of follow-up.

If the adjuvant use of L-PRF is analyzed with other treatments, for example, laser therapy or BMP-2, higher percentages of complete resolution of the lesion are obtained when it is used in combination with these (20,27). These two studies, moreover, are considered with the highest quality according to the JBI scale (Supplement 1; Supplement 2). In the trial by Park *et al*. (20), a sample of 55 patients was collected, which was divided into two groups, applying L-PRF as an adjunct therapy to both. The difference between one group and another would be the association, in addition, of BMP-2. As results, they obtained that the study group (L-PRF and BMP-2) presented a complete resolution of 96% compared to 88% using only therapy with L-PRF. However, as limitations, the non-existence of a control group stands out, due to the severity of the disease and its low prevalence, and that a group using only BMP-2 as treatment was not made to establish the comparison (20). There are no studies in the scientific literature that analyze the result of treatment using only BMP-2, so it cannot be ruled out that the result obtained by Park *et al*. (20) is mainly due to the synergistic action of both compounds (L-PRF and BMP-2) and that without the L-PRF such a high coating rate would not happen. The authors themselves conclude that fibrin acts as a support matrix for BMP-2, progressively releasing itself in the lesion, stimulating both soft tissue coverage and bone remodeling.

On the other hand, the study by Tenore *et al*. (27) also establishes several treatment groups: the first treated following the usual protocol (antibiotics, antiseptics and surgical debridement) with L-PRF and laser as adjunctive therapy, the second treated with antibiotics, antiseptics and surgery; and the third group treated only with antibiotics and laser. Of the three groups, the highest percentage of complete resolution is reached by the group that uses L-PRF and laser, followed by the one that uses the usual protocol and, lastly, the conservative approach with only antibiotics and laser. Laser treatment was similar in both cases and two sessions a week were performed for four weeks (27). Unlike the previous study, in this case the use of the laser alone is analyzed, but not with surgery, so it cannot be stated that the use of L-PRF is the only factor that enhances the coverage of the lesion (20,27). The last of the studies that used an additional treatment of L-PRF and the usual recommended protocol was that of Sahin *et al*. (25) where laser was used for biostimulation, reaching a complete resolution percentage of 100%. In the study by Momesso *et al*. (36), where the use of low-grade laser as the only treatment has been analyzed, in comparison with the traditional laser (Er:YAG) associated with surgery or with medical treatment (antibiotics and antiseptics), it has been reached the conclusion that laser with traditional surgery provides a greater number of patients with total coverage (around 90%), followed by laser with surgery and low-grade laser (88.2%), low-grade laser and surgery (73.6%), only surgery (69.3%), only low-grade laser (43%) and lastly, the use of only antibiotics and antiseptics (18%).

The rest of the included studies used only L-PRF (21-24,26,28) as adjuvant treatment. In two studies where the lowest percentages of lesion coverage were achieved, the patient sample was under treatment with nitrogenated bisphosphonates and denosumab (24,26). In Law *et al*. study where 75% of coverage was obtained, the patients were women and the lesions were located only in the mandible.

It is important to note that recent studies have shown its effectiveness not only in the treatment of pre-established disease, but also in preventive situations, in patients undergoing angiogenic or antiresorptive treatment who are going to treat with dentoalveolar surgery. This was already being done with other compounds' plasma derivatives (plasma rich in growth factors, platelet-rich fibrin), with satisfactory results (37,38). In the study by Asaka *et al*. (38), the coverage of the lesion is analyzed in patients undergoing treatment with BP who extractions were carried out. Tooth extractions are the main triggering factor for MRONJ reported in most studies (3,5,11,33,34). To do this, they established two groups, PRF group and control group, finding statistically significant differences in relation to the quick coverage of the lesion in patients who received PRF (38). In the study by Sahin *et al*. (25), where L-PRF was specifically used, a percentage of lesion coverage was obtained without the appearance of MRONJ in 100% of the subjects, with a reasonable sample and follow-up.

In established MRONJ, treatment with surgery is and has always been considered a good alternative, however, the adjuvant use of L-PRF as an adjunct therapy (alone or in combination with other therapies) provides extra benefits to achieving a wound healing of the injury in less time and with fewer comorbidities (20-28). L-PRF is an autologous treatment whose production is standardized in the literature, cheap and easy to manufacture with few resources. We can affirm that it is not only a compound that provides benefits in the treatment of MRONJ, but what is more important and fundamental, that constitutes a treatment strategy in the disease primary prevention. There are few studies in the literature that specifically use this compound for prevention in risk patients and situations, although, given its characteristics and easily preparation, it would be interesting to address this topic in greater depth in the coming years.

Finally, we would like to point out a series of limitations of the present meta-analysis. A very restricted number of clinical trials could be found. The majority of studies had sample sizes limited, at the same time the lack of control groups due to the heterogeneity of the primary literature, avoided the use of other measures different from prevalence to analyze the effect. This fact limited the possibility of comparing the effectiveness with other surgical, non-surgical or mixed protocols in a quantitative way.

In addition, the study protocols were heterogeneous and MRONJ cases were subject to many diverse factors, such as differences in the type of drugs used, underlying diseases, duration of drug administration, different triggering factors, comorbidities, follow-up and definitions of success. Taking all this information together, the generalization of the results obtained by this study must be taken cautiously.

## Conclusions

L-PRF alone or in combination with other therapies in the treatment of MRONJ achieved percentages of complete lesion resolution of 94.3%. In studies where L-PRF was used concomitantly with other therapies, and the effectiveness of the other therapy alone is analyzed, L-PRF has been shown to achieve higher percentages of resolution.
